# Study on Microstructural Evolution of DP Steel Considering the Interface Layer Based on Multi Mechanism Strain Gradient Theory

**DOI:** 10.3390/ma15134590

**Published:** 2022-06-29

**Authors:** Qianduo Zhuang, Zhenming Yue, Lingxiao Zhou, Xihang Zhao, Jiashuo Qi, Xinrui Min, Zhongran Zhang, Jun Gao

**Affiliations:** School of Mechanical, Electrical and Information Engineering, Shandong University at Weihai, Weihai 264209, China; 202017439@mail.sdu.edu.cn (Q.Z.); yuezhenming@sdu.edu.cn (Z.Y.); 201900800029@mail.sdu.edu.cn (L.Z.); 202017438@mail.sdu.edu.cn (X.Z.); qjs@mail.sdu.edu.cn (J.Q.); 201816481@mail.sdu.edu.cn (X.M.); shdgj@mail.sdu.edu.cn (J.G.)

**Keywords:** microstructures, geometrically necessary dislocations, local hardening, topology optimization, dual-phase steel

## Abstract

A multi-mechanism constitutive model is proposed in this paper to better describe the effect of the local hardening behavior of the interface layer on the mechanical heterogeneity of dual-phase (DP) steel. The constitutive equations considering the geometrically necessary dislocations (GNDs) and back stress at grain level and sample level were established. Based on the finite element simulation results, the influences of local hardening and microstructure characteristics on the strain–stress evolution, statistical storage dislocations, GNDs, and back stress of DP steel were studied and discussed. Due to the local hardening effect, the ferrite phase was treated as an inhomogeneous matrix reinforced by some small islands of martensite in the simulation. The simulation results show that the thickness of the interface layer has a significant effect on the macroscopic hardening property of DP steel, while the number of interface layers has little effect. Meanwhile, the GNDs and back stress at the grain level also have little effect on the strengthening of DP steel. The contribution of GNDs at the sample level to the flow stress is about 47%.

## 1. Introduction

Advanced high strength steels are being developed rapidly with requirements of weight reduction and high crashworthiness. Due to the heterogeneous microstructure of dual-phase (DP) steel, DP steel has high ultimate tensile strength and good ductility [1,2]. The flow behavior of DP steel depends on many aspects, including the martensite distribution, grain orientation, chemical composition, etc. DP steels are low-carbon alloyed steels characterized by their multiphase structures. They have a soft ferrite matrix phase and an embedded hard martensite. They behave like composites in which the ferrite matrix ensures good formability and the martensite acts as a reinforcement.

Various methodologies have been used to predict and quantify the forming processes and the work hardening behaviors of metals and composites [3,4,5]. However, it is still difficult and expensive to quantitively reveal the relationship between the microstructure and the mechanical properties through experiments. Therefore, many researchers have chosen to investigate the flow behavior of DP steels by numerical simulation. Representative volume element (RVE) has been proved to be an efficient method that can represent well the multi-scale forming behavior of the multiphase material based on the rebuilt microstructure model [6,7,8,9,10].

The mechanical properties and microstructure characteristics of martensite and ferrite phases, such as grain size, phase content, and morphology of the martensite and ferrite, determine the plasticity and fracture behaviors of DP steels [11,12,13,14]. To accurately investigate the plasticity and fracture behaviors of DP steels, Asik et al. [14] applied a strain gradient enhanced crystal plasticity model to investigate the effect of the martensite distributions (zonal and random) on damage evolution with RVE. The effect of microstructure characteristics on the plasticity, strain localization, and strain mechanisms was investigated and studied by Hou et al. [15]. The electron backscatter diffraction (EBSD) image mapping methodology can be used for the RVE geometrical modelling [16]. The phase properties can be obtained by the inverse simulation of nanoindentation experiments. Experimental and simulation results demonstrate a good agreement on the mechanical properties display for DP steel with the nanoindentation method. It was found that the ferrite region near to the martensite is the most critical factor affecting the strain localization and ductile fracture evolution.

In recent years, much research has been conducted on the material mechanical properties at the ferrite–martensite interface, which can be considered as the phase affecting strength and ductility [17]. Kadkhodapour et al. [18] investigated the relationship between the residual stresses and the yield behaviors of DP steels by considering their microstructure evolution. It was found that the dislocation density accumulated at the interface results in local hardening and the microstructure changes mainly at the interface. The influence of the ferrite phase on the macroscopic behavior of DP steel was studied by finite element methodology which considers the hardness variation of the ferrite phase. The same results were also validated by Ramazani et al. [19], who numerically defined a high-GND-density zone around the martensite grain, while the zone can also be regarded as a pre-strained zone induced by the austenite-to-martensite transformation. Meanwhile, mesoscale finite element simulations were conducted with the assumption of the existence of hard zones around the interface. The results represented the macro stress precisely and are consistent with the experimental responses [18,19]. However, the hardening mechanism introduced by the GNDs, statistical storage dislocations (SSDs), the back stress, and other micro-mechanisms is still unclear and is attracting a lot of attention.

**Figure 1:**
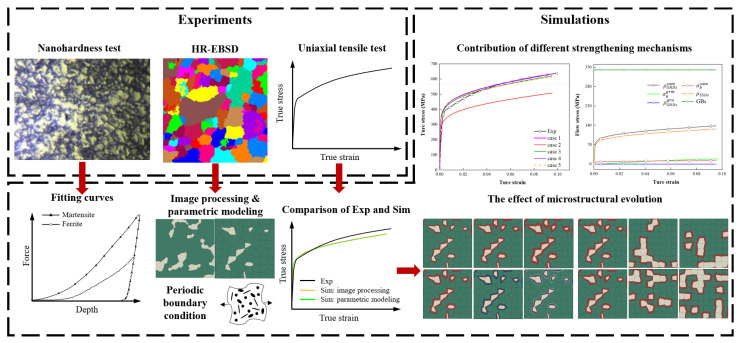
The research scheme involving the experiments and simulations.

In this study, the interface layer with different microstructures was considered, and the hardening flow behavior of ferrite and martensite was calculated and analyzed based on the multi-mechanism strain gradient theory. Then, the RVE with defined microstructures was numerically tested under the uniaxial tensile loading. The purpose of this paper was to investigate the effect of microstructure on the overall plastic behavior, in particular the individual contribution of different strengthening mechanisms to the overall stress–strain response. The research scheme of this study is shown in Figure 1.

## 2. Material, Experimental Procedure, and Observations

### 2.1. Material and Multi-Scale Experiment

In this study, DP600 steel sheet with thickness 1.2 mm was chosen for the tests. The phase ratios of materials were 17.2% martensite and 82.8% ferrite. Their mechanical properties were obtained with the help of nano-indentation tests [20]. Three samples were tested to reduce the random errors and the chemical composition of DP600 is given in Table 1. The annealing process was carried out to enhance its mechanical response. The uniaxial tensile tests were performed following the rolling direction to obtain the macroscopic hardening behavior. The two-dimension EBSD and nano-indentation tests were carried out to obtain the microstructure parameters.

### 2.2. Microstructure Observation

**Figure 2:**
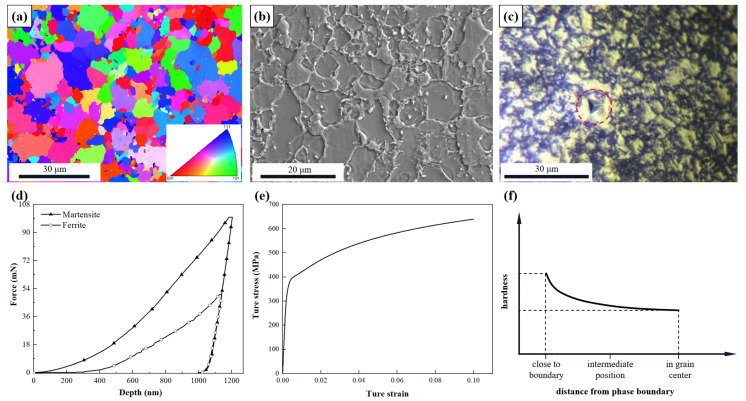
Multi-scale characterizations of DP-steel: (**a**) EBSD map; (**b**) SEM micrograph; (**c**) metallographic nano-indentation test; (**d**) mechanical response of ferrite and martensite during nano-indentation; (**e**) single tensile curve with specimen dimensions. (**f**) Schematic diagram of decreasing hardness of ferrite phase away from the interface.

The texture and microstructure are the key parameters that affect the mechanical properties of structural steels. Figure 2a gives the grain distribution with the EBSD map. Figure 2b shows the micrograph observed by scanning electron microscope (SEM) with the small ferrite grains surrounded by the large martensitic grains. Figure 2c shows the results of the Berkovich indenter pressed into the grains of the DP steel. Figure 2d shows the mechanical response curves of ferrite and martensite obtained by the nano-indentation test. With the help of overall geometrical model simulation of the nano-indentation tests, the mechanical response of DP600 can be obtained with one inversed parameter identification methodology. Figure 2e shows the final obtained stress–strain of DP steel. In all cases, much higher hardening behavior can be observed near the interfaces. From previous reports [18,21], the volume fraction of GNDs appears higher in the small-grain of ferrite whose grain sizes are approximately 2.0–5.0 μm. The experiment obtaining the relationship between the ferrite hardness and the distance to the phase boundary is shown in Figure 2f.

## 3. Microstructural Modeling and Numerical Implementation

### 3.1. Microstructure-Based Modelling with Two Methods

**Figure 3:**
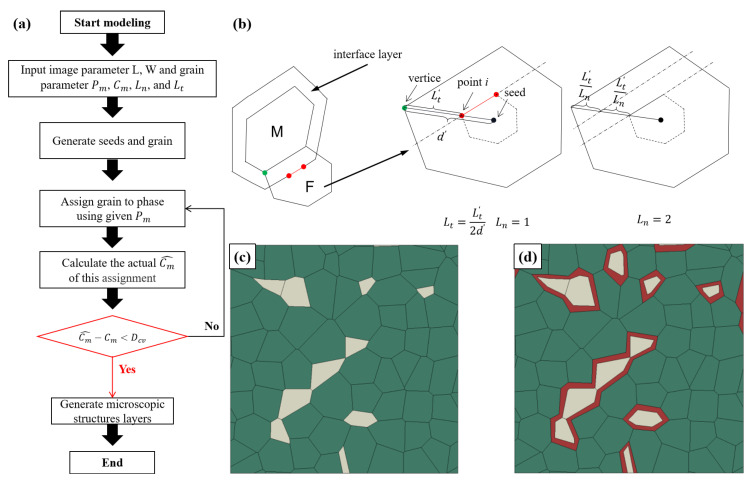
(**a**) The flowchart of the microstructure-based model; (**b**) schematic diagram of interface layer parameters; RVEs without interface layer (**c**) and with interface layer (**d**) in ABAQUS.

The flowchart process of establishing the microstructure-based model is shown in Figure 3a, in which the hardening effect of the interface layer is considered. $P_{m}$ and $C_{m}$ are used to restrict the distribution of ferrite and martensite [22]:

(1) $$P_{m}=\frac{A_{m}}{A_{t}},\;C_{m}=\frac{2L_{MM}}{2L_{MM}+L_{FM}}$$

where the $A_{m}$ is the volume of the martensite phase and $A_{t}$ is the total volume. $L_{MM}$ denotes the total boundary length of martensite–martensite grains and $L_{FM}$ denotes the boundary length of martensite–ferrite grains.

$L_{n}$ and $L_{t}$ are the number of layers and the percentage of the layer thickness to the total thickness of grain. They are first produced to evaluate the behavior of the interface layer. Fillafer [22] modified the seed sequence through Halton (quasi-random) sequences so that the particles had a very poor aspect ratio, i.e., the minimum internal angle of the particles was less than about 15°.

A phase assignment algorithm based on topology optimization is used to allocate the ferrite and martensite phases with given phase parameters $P_{m}$ and $C_{m}$. Grains are generated through Voronoi using the modified seed sequence. For Voronoi tessellations and phase distribution mode, the $P_{m}$ and $C_{m}$ can be calculated by analyzing for each grain. Thus, $P_{m}$ and $C_{m}$ are implemented through multiple iterations until the error between the actual and the given parameters is small enough (usually an error of less than 1% is considered acceptable).

Proceed as follows. Take points proportionally from the seed to the six vertices and connect each point in turn to generate a layer. $L_{n}$ is the number of equal parts from the vertex to the point *i* as in Figure 3b. At the same time, to standardize the thickness of boundary layers between different martensite phases, we introduce the concept of standard layer thickness $L_{t}$ as:

(2) $$L_{t}=\frac{L_{t}^{\prime }}{d^{\prime }}$$

where $L_{t}^{\prime }$ is the distance from the vertex to the point *i* and $d^{\prime }$ is the distance from the seed to the vertex.

Because periodic microstructures have favorable numerical properties in the context of computational homogenization [23], these seeds are repeated three times in the X and Y directions to ensure the periodicity of Voronoi.

In general, RVE size has a significant effect on the simulation results. The size of the representative RVE must be large enough to represent all the microstructure characteristics while remaining small enough to be considered statistically uniform when calculating validity. In the study of Ramazani et al. [24], it was concluded that the minimum acceptable size of DP steel RVE is 24 microns. The 2D model is set to 50 microns by 50 microns. Figure 3c,d show the standard Voronoi Mosaic generating 50 grains based on the same seed set and the Voronoi Mosaic with layered ferrite phase. Yuliang Hou et al. [15] studied the effect of phase distribution topology on the plastic behavior of dual-phase steels using 2D RVE with a size of 25 µm × 25 µm.

### 3.2. Load, Periodic Boundary Condition, and Meshing

**Figure 4:**
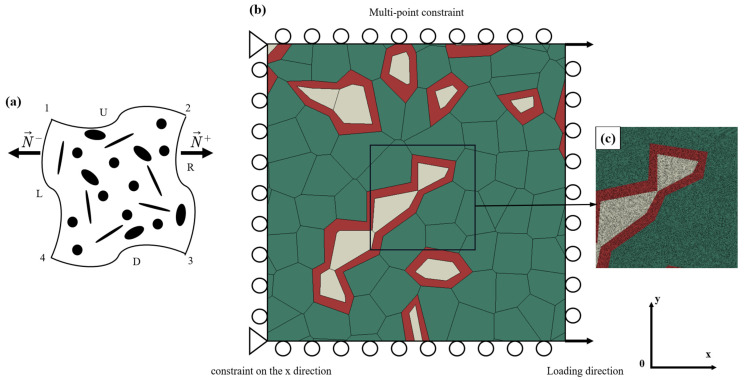
(**a**) Comparison of different boundary conditions. Schematic diagram of (**b**) RVE single pull load condition. (**c**) Meshed RVE.

Because the periodic boundary condition was the most efficient in terms of convergence rate as the RVE size increases [25], the periodic boundary condition was applied to the microstructure mode. Xia et al. [26] proposed a unified displacement differential periodic boundary condition. As shown in Figure 4a, the periodic boundary condition constraint equation of the finite element model is defined as:

(3) $$x_{U}=x_{D}+x_{4}-x_{1}\;x_{R}=x_{L}+x_{2}-x_{1}\;x_{3}=x_{2}+x_{4}-x_{1}$$

where $x_{U}$, $x_{R}$, $x_{D}$, and $x_{L}$ represent the displacement of the upper, right, lower, and left boundaries in the deformation process, and $x_{1}$, $x_{2}$, $x_{3}$, and $x_{4}$ represent the displacement of points 1, 2, 3, and 4 in the deformation process, respectively.

In Figure 4a, the microstructure model of DP steel with ferrite phase stratification was drawn in the XY Cartesian coordinate system. Fixed constraints were applied to reference points 1 and 4, and the constraints were set as 3 degrees of freedom fixed. Reference points 2 and 3 apply a node-displacement load, whose constraint is set to translate only with the $U_{x}$ direction load.

Shown in Figure 4c is the 2D structural model of DP steel meshed with 8-node biquadratic plane strain quadrilateral type (CPE8) elements. Eight integral points are applied to each element in the structural model. The average mesh size of DP steel is 200 nanometers, which is used to produce fine mesh. In this analysis, each 2D model structure of the DP steel yielded 280,000 elements. The simulation results of displacement $U_{x}$ and reaction force reflected by reference points 3 and 4 are described and discussed in detail later.

### 3.3. Flow Behaviors of Ferrite and Martensite Phases

For an elastoplastic body, the total strain rate ${\dot{\varepsilon }}_{ij}$ is considered to consist of an elastic part ${\dot{\varepsilon }}_{ij}^{e}$ and a plastic part ${\dot{\varepsilon }}_{ij}^{p}$ in the elastoplastic constitutive model as

(4) $${\dot{\varepsilon }}_{ij}={\dot{\varepsilon }}_{ij}^{e}+{\dot{\varepsilon }}_{ij}^{p}$$

The relationship between the elastic strain rate and the stress rate is given by Hooke’s law as

(5) $${\dot{\varepsilon }}_{ij}^{e}=\frac{1}{2\mu }{\dot{S}}_{ij}+\frac{{\dot{\sigma }}_{kk}}{9K}\delta_{ij}$$

where *μ* is the shear modulus, *K* is the bulk modulus, ${\dot{\sigma }}_{kk}$ is the hydrostatic stress rate and $\delta_{ij}$ is Kronecker’s symbol. The deviatoric stress rate ${\dot{S}}_{ij}$ is given as:

(6) $${\dot{S}}_{ij}={\dot{\sigma }}_{ij}-\frac{{\dot{\sigma }}_{kk}\delta_{ij}}{3}$$

The plastic strain rate ${\dot{\varepsilon }}_{ij}^{P}$ is determined by the deviatoric stress according to the *J_2_*- flow theory as [27]

(7) $${\dot{\varepsilon }}_{ij}^{p}=\frac{3{\dot{\varepsilon }}^{p}}{2\sigma_{e}}{\dot{S}}_{ij}$$

where ${\dot{\varepsilon }}^{p}=\sqrt{2{\dot{\varepsilon }}_{ij}^{p}{\dot{\varepsilon }}_{ij}^{p}/3}$ is the effective plastic strain rate, and $\sigma_{e}=\sqrt{3\sigma_{ij}^{\prime }\sigma_{ij}^{\prime }/2}$ is determined to be the effective stress according to a power-law viscoplastic formulation:

(8) $${\dot{\varepsilon }}^{p}={\dot{\varepsilon }}_{0}{\left(\frac{\sigma_{e}}{\sigma_{f}}\right)}^{m}$$

where ${\dot{\varepsilon }}_{0}$ is the reference strain rate, *m* is the rate-sensitivity which usually takes a large value, $\sigma_{f}$ is the flow stress controlling plastic deformation.

According to Kok [28], ${\dot{\varepsilon }}_{0}$ is replaced by the effective strain rate $\dot{\varepsilon }$ by eliminating strain rate and time dependence, and $\dot{\varepsilon }$ is given as

(9) $$\dot{\varepsilon }=\sqrt{\frac{2}{3}{\dot{\varepsilon }}_{ij}^{\prime }{\dot{\varepsilon }}_{ij}^{\prime }}$$

where ${\dot{\varepsilon }}_{ij}^{\prime }$ denotes the deviatoric strain rate:

(10) $${\dot{\varepsilon }}_{ij}^{\prime }={\dot{\varepsilon }}_{ij}-\frac{{\dot{\varepsilon }}_{kk}\delta_{ij}}{3}$$

For DP steel materials, the flow stress which considers dislocation density as the internal variable to control strain hardening is given as [29,30,31]

(11) $$\sigma_{f}=\sigma_{y}+M\alpha \mu \theta \sqrt{\rho }+\sigma_{b}$$

where $\sigma_{y}$ is the initial yield stress, $M\alpha \mu \theta \sqrt{\rho }$ which expresses the strain hardening resulting from dislocations is given by the Taylor hardening law [32,33], where *ρ* represents dislocation density, *M* is the Taylor factor, α is a constant that differs from the material, θ is the magnitude of the Bergers vector, $\sigma_{b}$ is the back stress and reflects kinematic hardening, while the other two terms on the right side of Equation (11) reflect isotropic hardening.

Generally, the isotropic hardening of deformed metal is considered to be caused only by SSDs. However, the heterogeneous deformation of the two phases (that is, the ferrite phase and martensite phase) in DP steel induced GNDs. Hence, the total dislocation density could be divided into three parts considering sample-level and grain-level GNDs [34] as

(12) $$\rho =\rho_{SSDs}+\rho_{GNDs}^{sam}+\rho_{GNDs}^{gra}$$

where $\rho_{SSDs}$, $\rho_{GNDs}^{sam}$, $\rho_{GNDs}^{gra}$ are the density of SSDs, sample-level GNDs, and grain-level GNDs, respectively.

In addition, the back stress can be divided into the following two parts:

(13) $$\sigma_{b}=\sigma_{b}^{sam}+\sigma_{b}^{gra}$$

where $\sigma_{b}^{sam}$ and $\sigma_{b}^{gra}$ are sample and grain levels back stresses, respectively.

Ultimately the flow stress is expressed below while the yield and strain-hardening behaviors of materials depend on their grain size [35].

(14) $$\sigma_{f}=\sigma_{y}+M\alpha \mu \theta \sqrt{\rho_{SSDs}+\rho_{GNDS}^{sam}+\rho_{GNDs}^{gra}}+\sigma_{b}^{sam\;}+\sigma_{b}^{gra}$$

Hall–Petch successfully expressed the yield stress based on the grain size, given as the Hall–Petch formula [36,37],

(15) $$\sigma_{Y}=\sigma_{0}+\frac{k_{HP}}{\sqrt{d}}$$

where $\sigma_{0}$ is the lattice friction stress and $\frac{k_{HP}}{\sqrt{d}}$ is the strengthening effect from GBs. $k_{HP}$ is the Hall–Petch slope, *d* is the grain size.

An evolution of SSDs density is given as:

(16) $$\frac{\partial \rho_{SSDs}}{\partial \varepsilon^{P}}=M\left[\frac{k_{mfp}^{g}}{bd}+\frac{k_{mfp}^{dis}}{b}\sqrt{\rho_{SSDs}+\rho_{GNDs}}-k_{ann}^{0}{\left(\frac{{\dot{\varepsilon }}^{P}}{{\dot{\varepsilon }}_{ref}}\right)}^{-\frac{1}{n_{0}}}\rho_{SSDs}-{\left(\frac{d_{ref}}{d}\right)}^{2}\rho_{SSDs}\right]$$

The grain-level GNDs in homogeneous polycrystals are the pileup dislocations that accommodate slip discontinuities between grains. The sample-level GNDs are the dislocations distributed in polycrystalline clusters in the integration point. The back stress is determined by the corresponding GNDs [38].

At the sample-level, GNDs are generated by the deformation incompatibility of each phase. Nye [39] and Ashby [34] used the effective plastic strain gradient $\eta^{P}$ to calculate GND density.

(17) $$\rho_{GNDs}^{sam}=\bar{r}\frac{\eta^{P}}{b}$$

where $\bar{r}$ is the Nye factor. According to Gao et al. [40], effective plastic strain gradient $\eta^{P}$ is defined as:

(18) $$\eta^{P}=\sqrt{\eta_{ijk}^{P}\eta_{ijk}^{P}/4}$$

where the third-order tensor $\eta_{ijk}^{P}$ is given as:

(19) $$\eta_{ijk}^{P}=\varepsilon_{ik,j}^{P}+\varepsilon_{jk,i}^{P}-\varepsilon_{ij,k}^{P}$$

Then tensor for plastic strain is given as:

(20) $$\varepsilon_{ij}^{P}=\int {\dot{\varepsilon }}_{ij}^{P}dt$$

where indices *i* and *j* correspond to x and y coordinate directions, respectively.

According to Bayley et al. [41] and Zhao et al. [38], the effective back stress $\sigma_{b}^{sam}$ can be calculated in a von Mises form as:

(21) $$\sigma_{b}^{sam}=\sqrt{3{\left(\frac{\partial \rho_{GNDS}^{sam}}{\partial y}\right)}^{2}}\frac{\mu bR^{2}}{8(1-v)}$$

where *R* is the integral circular domain within which the GNDs contributes to back stress, *v* is Poisson’s ratio, and the nondimensional coefficient *D* is obtained through consideration of ρ [42].

At the grain-level, GNDs pile up in front of the grain boundaries (GBs), and their density can be calculated on an average basis [38]

(22) $$\rho_{GNDs}^{gra}=\frac{N}{d^{2}}$$

where *N* is the total number of piled-up dislocations and *d* is the grain size. According to Sinclair et al. [43] and Zhao et al. [38], the evolution of *N* with plastic strain can be modified as:

(23) $$\frac{\partial N}{\partial \varepsilon^{P}}=N_{\Delta }\left(1-\frac{N}{N^{*}}\right)$$

where, $N^{*}$ is the dislocation saturation number that GBs can maintain, and $N_{\Delta }$ is the initial evolution rate of pileup dislocation. In larger particles, the vicinity of GBs provides more space for pileups. The saturation number of stacking dislocation $N^{*}$ is also positively correlated with the grain size *d*. However, due to the lack of microscopic measurements and models, it is difficult to obtain accurate correlation. This paper refers to the hypothesis of Zhao et al. [38] that $N^{*}$ and *d* are linear, i.e.,

(24) $$N^{*}=\lambda d+N_{extra\;}$$

where $N_{extra}$ is constant and *λ* is the proportional coefficient. The study of Zhu et al. [31] also showed that the larger the particle size, the larger was the $N_{\Delta }$. This paper adopts the linear relation between *d* and $N_{\Delta }$ to deal with this relation, namely:

(25) $$N_{\Delta }=k_{N}d+N_{A}$$

A pileup of GND inside the particles generates back stress that inhibits subsequent dislocation movement further towards GBs. According to Hirth et al. [44], if the N pileup edge distributions are double-ended, then the induced back stress at grain level is calculated as:

(26) $$\sigma_{b}^{gra}=\frac{M\mu bN}{\pi (1-v)d}$$

where the Taylor factor *M* is used to connect the macroscopic and shear stresses of the slip system.

In the following sections, the user material subroutine is used to implement the model into the finite element software ABAQUS (6.14) [44] to invert the material parameters of martensite and ferrite in DP Steel.

### 3.4. Inverse Identification of Constitutive Parameters

In the constitutive model, the magnitude of Burgers vector *b*, the Hall–Petch constant $k_{HP}$, and other constitutive parameters have physical significance. The values of these parameters are almost constant and can be obtained from the literature. Other constitutive parameters of DP steel were calibrated by simulating the tensile properties of DP steel. These parameters can be obtained by the following methods.

**Figure 5:**
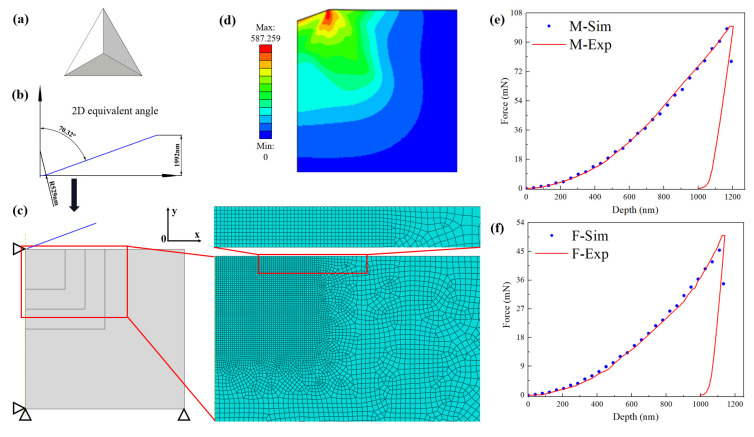
Finite element model and fitting curves for nano indentation simulations: (**a**) Berkovich indenter; (**b**) top rounding of Berkovich indenter; (**c**) 2D axisymmetric finite element model; (**d**) stress distribution of 2D model; fitting curves of (**e**) martensite and (**f**) ferrite.

As shown in Figure 5a, the Berkovich indenter is a triangular pyramid shape of a regular tetrahedron. As shown in Figure 5b, to simplify the simulation process and avoid mesh penetration in finite element simulation, Berkovich’s equivalent conical indentor with a rounded tip (α = 70.3°) was equivalent in the two-dimensional model. Meanwhile, combined with the research results of Li et al. [45], the radius of the conical corner arc was set as 529 nm. Figure 5c presents the schematic diagram of the finite element model after 2d axisymmetric modeling and grid division. The horizontal displacement of the nodes on the symmetry axis of the sample and the axial displacement of the nodes on the lower boundary are set to zero. The reference point of the rigid head can only move in the vertical direction, and it has only one degree of freedom downward. Figure 5d shows the simulated stress–strain cloud diagram after applying a fixed displacement load. In Figure 5e,f, the load-displacement comparison curves of the simulation and experiment of the two-dimensional nanoindentation model are presented. The shear modulus *μ* can be calculated by the relationship of *μ* with the modulus of elasticity and Poisson’s ratio. Table 2 shows the mechanical parameters of the martensite and ferrite.

## 4. Result and Discussion

### 4.1. Validation of the Microstructural RVE on Mechanical Behaviors of DP Steel

**Figure 6:**
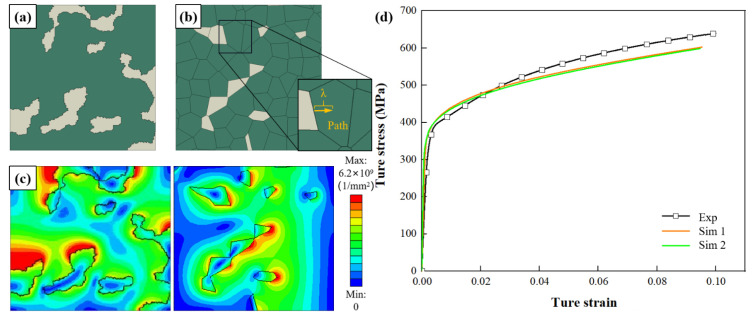
Result distribution and mechanical properties of different RVEs based on (**a**) image processing and (**b**) parametric modeling (gray and green cells represent martensite and ferrite grains, respectively.): (**c**) GNDs (*ε* = 10.0%); (**d**) comparison of experiment and simulation for tensile test.

RVEs were generated based on the image processing and parametric modeling of DP600. The control parameters of the DP steel are $P_{m}$ = 17.2% and $C_{m}$ = 32.0%. RVEs and the corresponding finite element models are shown in Figure 6a,b respectively. Then the flow characteristics of the component phase were further studied.

Figure 6c shows that GNDs mainly occur at the boundary of ferrite–martensite. Figure 6d shows good agreement between experimental and numerical tensile stress–strain relationships for DP steels. To systematically understand the deformation process of DP steel, it is necessary to investigate the effects of $L_{t}$, $L_{n}$, $P_{m}$, and $C_{m}$, on the microscopic and macroscopic plasticity behaviors.

### 4.2. Effect of the Thickness of the Interface Layer

**Figure 7:**
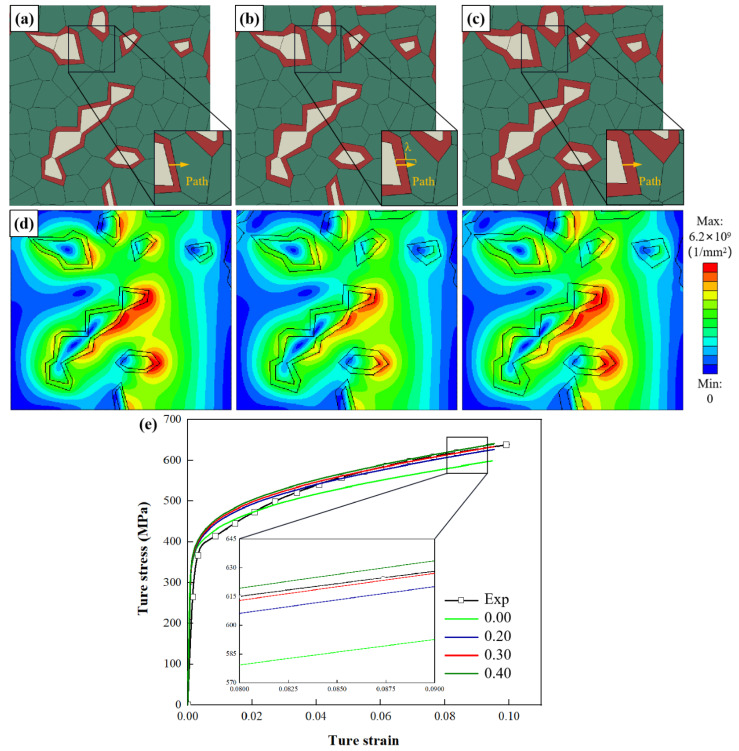
RVEs generated with *P_m_* = 17.2% and C*_m_* = 0.32, and by varying the parameter *L_t_* as (**a**) *L_t_* = 0.20, (**b**) *L_t_* = 0.30, (**c**) *L_t_* = 0.40; (**d**) GND density distribution of RVEs (*ε* = 10.0%); (**e**) comparison of experimental and numerical stress–strain curves.

According to the results of nano-indentation tests, the strength of the interface layer is 35% higher than that of the ferrite matrix. Figure 7a–c shows three different layer thickness RVEs of DP steel materials with interface layer layers of one layer. Figure 7d shows the geometric dislocation density distribution when the macroscopic strain is 10%. Due to the increase of the boundary layer, there are soft and hard differences in ferrite, resulting in uneven dislocation distribution.

Figure 7e shows the relationship between stress and strain under tensile path. It can be seen that after increasing the thickness of the interface layer, the thicker the interface layer, the greater the mechanical response, which is consistent with the results of other authors [18]. When the thickness of the layer is 30%, the macroscopic tensile behavior agrees best with the experiment, and the hypothesis in the model seems to be realistic. It is also evident that a layer thickness of 30% of the one-layer model using this material model provides good accuracy through precise parameter selection. Therefore, this follow-up study will use a 30%-layer thickness model to investigate the influence of layer number.

**Figure 8:**
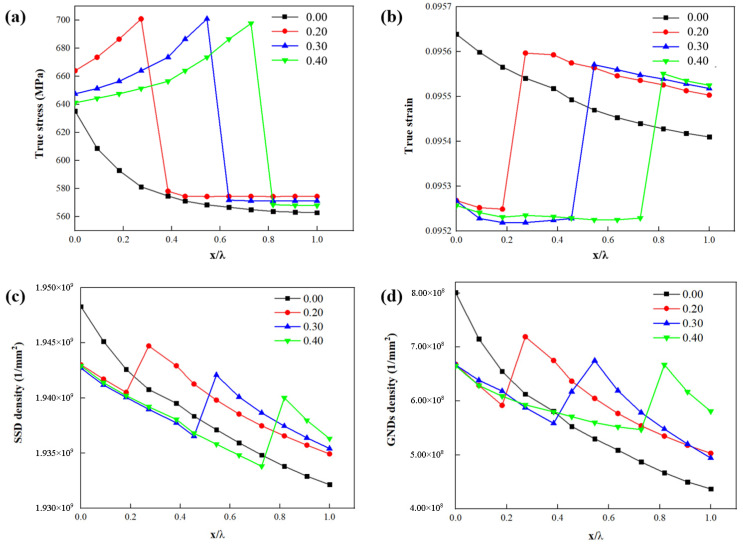
Influence of parameter *L_t_* on the distribution of (**a**) stress *σ*, (**b**) strain *ε*, (**c**) SSDs and (**d**) GNDs along four paths where the four paths are marked in Figure 6b and Figure 7a–c. (x is the distance from the start of the path; λ is the total length of the path).

Figure 8a shows that the highest stress σ occurs at the boundary between the interface layer and ferrite. With the addition of interface layer thickness, the highest stress σ occurs at the boundary between the interface layer and ferrite. Figure 8b shows that the highest strain ε occurs at the boundary between interface layer and ferrite. With the addition of the interface layer, the strain ε decreases at the boundary between the martensite and the interface layer. The highest strain ε occurs at the border demarcating the interface layer and the ferrite.

Figure 8c shows that the highest SSDs occur at the border demarcating the martensite and interface layer. With the addition of interface layer thickness, the strain ε decreases at the boundary between the martensite and interface layer. The highest SSDs occur at the boundary between the interface layer and the ferrite. In addition, the peak value of SSDs decreases with layer thickness. Figure 7d shows that the GNDs distribute in both martensite and ferrite, and the maximum GND density appears at the border of interface layer and ferrite. Figure 8d shows that the peak value of GNDs between interface layers decreases with the increase of interface layer thickness.

### 4.3. Effect of the Number of the Interface Layer

**Figure 9:**
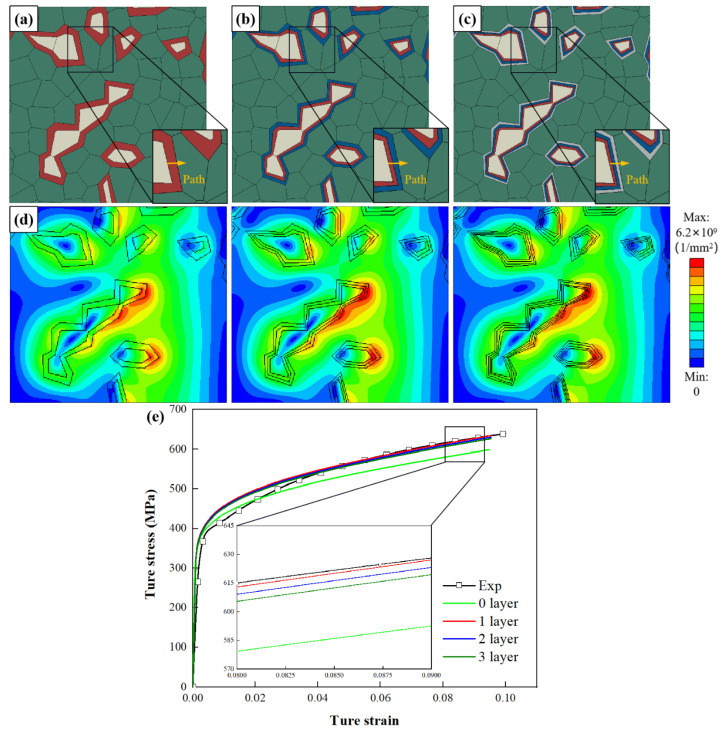
RVEs generated with *P_m_* = 17.2%, *C_m_* = 0.32 and *L_t_* = 0.30, and by varying the parameter *L_n_* as (**a**) *L_n_* = 1, (**b**) *L_n_* = 2, (**c**) *L_n_* = 3; (**d**) GND density distribution of RVEs (*ε* = 10.0%); (**e**) comparison of experimental and numerical stress–strain curves.

For the interface layer, a different number of layers is considered to check the accuracy of the model in macroscopic mechanical stress–strain behavior. Figure 9a–c shows three different layer number unit models of DP steel materials with a phase boundary layer thickness of 0.30. (Refer to the results of detailed nano-hardness tests on the microstructure of DP steel). When layered into two layers, the strength of the interface layer is thought to be 50% and 20% higher than that of the ferrite matrix. When stratified into three layers, the strengths of the interface layers were considered to be 50%, 35%, and 20% higher than that of the ferrite matrix. Figure 9e shows the comparison between the results of single pull simulation and experimental data for three different layers of DP steel materials with an interface layer thickness of 30%. The results showed good consistency which is consistent with the results of other authors [18]. With the increase of the number of layers, the macroscopic tensile properties are almost unchanged. Therefore, one-layer 30%-layer thickness interface layer model will be adopted in the subsequent study to investigate the influence of phase distribution topology.

**Figure 10:**
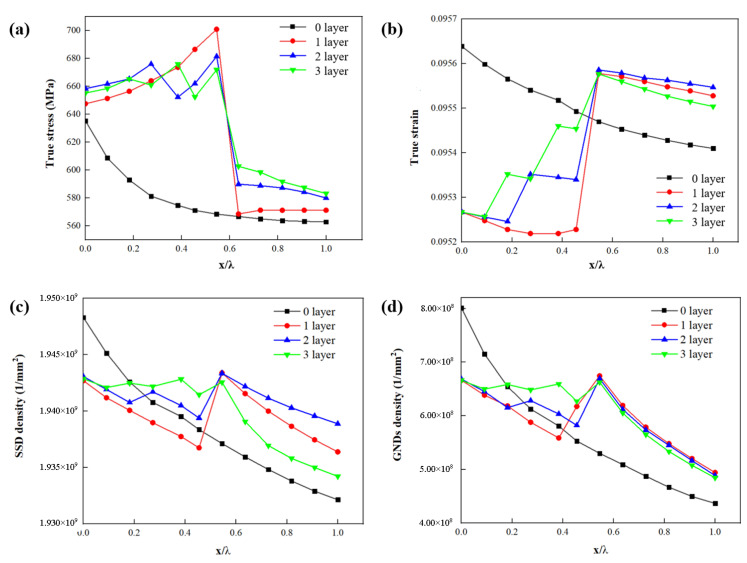
Influence of parameter *L_n_* on the distribution of (**a**) stress *σ*, (**b**) strain *ε*, (**c**) SSDs and (**d**) GNDs along four paths where the four paths are marked in Figure 6b and Figure 9a–c. (x is the distance from the start of the path; λ is the total length of the path).

Figure 10a shows that as the number of interface layers increases, stress σ peaks occur between the interface layers and ferrite. However, the highest stress σ occurs at the border demarcating the interface and the ferrite. Figure 10b shows that as the number of interface layers increases, the strain ε decreases at the ferrite and interface, and strain ε peaks occur between the interface layers. However, the highest strain ε occurs at the border demarcating the interface and the ferrite. Figure 10c shows that as the number of interface layers increases, the SSDs decrease at the martensite and interface, and SSD peaks occur between interface layers. However, the highest SSDs occur at the border demarcating the interface and ferrite. Figure 10d shows the distribution and evolution of GNDs. The peak value of GNDs exists between interface layers. As the number of layers increases, so does the number of peaks.

### 4.4. Effect of Martensite Phase Fraction

**Figure 11:**
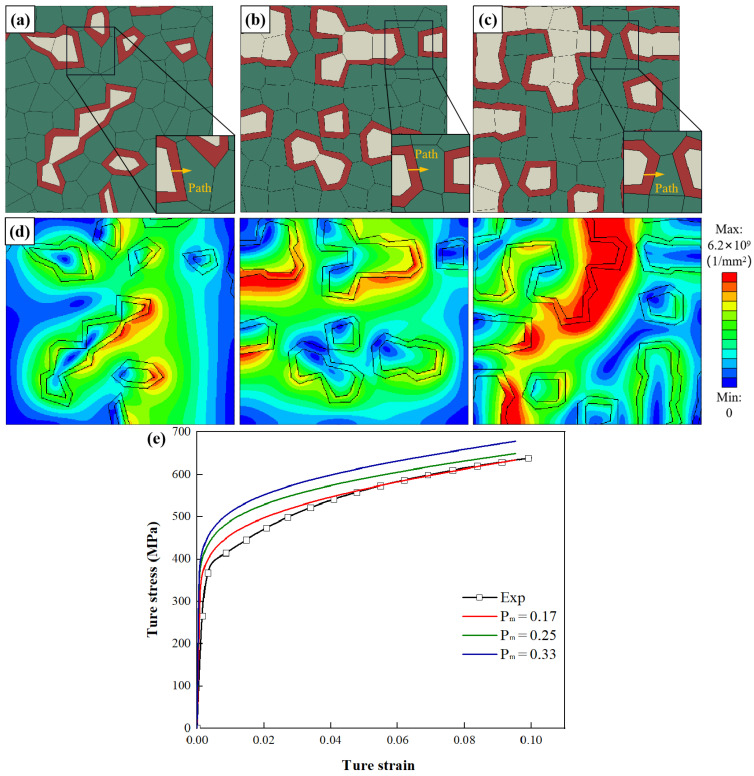
RVEs generated with *L_t_* = 0.30, *L_n_* = 1 and *C_m_* = 0.32, and by varying the parameter *P_m_* as (**a**) *P_m_* = 17.2%, (**b**) *P_m_* = 25.2%, (**c**) *P_m_* = 33.2%; (**d**) GND density distribution of RVEs (*ε* = 10.0%); (**e**) comparison of experiment and simulation for tensile test.

To investigate the influence of $P_{m}$ on the work hardening of DP steel, three RVEs are generated, with $C_{m}$ = 0.32 and with $P_{m}$ = 17.2%, 25.2%, and 33.2%, as shown in Figure 11a–c. Figure 11e shows the comparison of experiment and simulation for the tensile test. It was found that the flow stress was improved with the increase of $P_{m}$ which is also consistent with the findings of another study [24].

**Figure 12:**
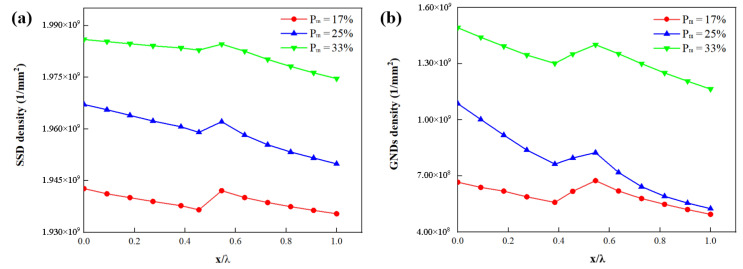
Influence of parameter *P_m_* on the distribution of (**a**) SSDs and (**b**) GNDs along four paths where the four paths are marked in Figure 11a–c. (x is the distance from the start of the path; λ is the total length of the path).

Figure 12a,b shows the distribution of SSDs and GNDs along three paths where the three paths are marked in Figure 11a–c. The local SSDs and GNDs increase with the parameter $P_{m}$. The density distribution of GNDs due to kinematic incompatibility within the structure is strongly influenced by the martensite volume fraction. These in turn affect its flow stress. When $P_{m}$ = 17%, the maximum density of GNDs is found. GND peaks occur between interface layers.

### 4.5. Effect of Martensite Phase Distribution

**Figure 13:**
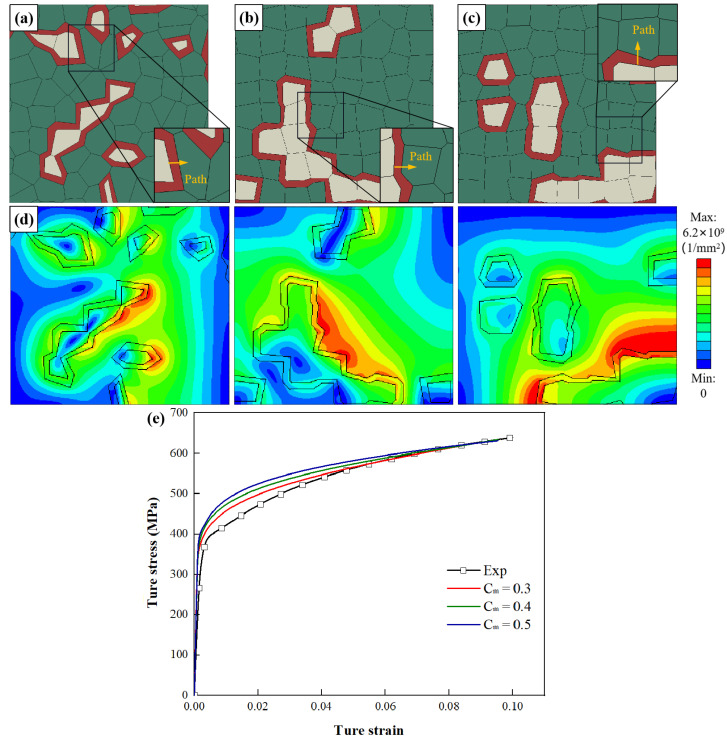
RVEs generated with *L_t_* = 0.30, *L_n_* = 1 and *P_m_* = 17.2%, and by varying the parameter *C_m_* as (**a**) *C_m_* = 0.32, (**b**) *C_m_* = 0.42, (**c**) *C_m_* = 0.52; (**d**) GND density distribution of RVEs (*ε* = 10.0%); (**e**) comparison of experiment and simulation for tensile test.

**Figure 14:**
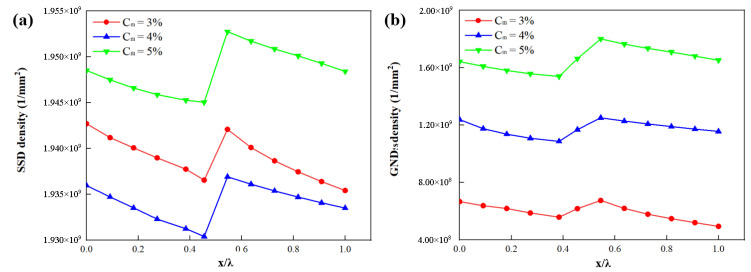
Influence of parameter *C_m_* on the distribution of (**a**) SSDs and (**b**) GNDs along four paths where the four paths are marked in Figure 13a–c. (x is the distance from the start of the path; λ is the total length of the path).

Figure 13a–c shows three RVEs with $P_{m}$ = 17.2%, and with $C_{m}$ equal to 0.32, 0.42, and 0.52. There is a slight difference between the flow stress curves produced by changing only $C_{m}$. With the increase of $C_{m}$, the higher the aggregation degree of martensite, the more GNDs are generated (Figure 13d). When the strain is less than 6%, the plastic behavior of DP steel is enhanced with the increase of $C_{m}$. However, when the strain gradually reaches 10%, the plastic behavior of DP steels with different $C_{m}$ becomes gradually equal. It can be seen from Figure 14 that the change of $C_{m}$ has no impact on the trend change of SSDs and GNDs. The local deformation is strongly influenced by the average distance between the martensite islands [14], while the location of the path has a great influence on the results. The strain level of the ferrite is significantly higher in the DP steels with $C_{m}$ = 0.5 compared to $C_{m}$ = 0.3. The local SSDs and GNDs increase with the volume fraction of $C_{m}$. The results showed good consistency which is consistent with the results of other authors [49].

### 4.6. Contribution of Strengthening Mechanisms

To quantify the contribution of $\rho_{GNDs}^{sam}$, $\sigma_{b}^{sam}$, $\rho_{GNDs}^{gra}$, $\sigma_{b}^{gra}$ and $\rho_{SSDs}$ to the global stress–strain response of DP steel under uniaxial tension, RVEs with $L_{t}$ = 0.30, $L_{n}$ = 1, $P_{m}$ = 17.2% and $C_{m}$ = 0.32 were used as the research subject. Five modeling scenarios were designed, and their contributions were separated, as shown in Table 3. “√” indicates that the factor is taken into account, and “×” indicates that its influence is eliminated. Case 1 includes all mechanisms. Radically, $\sigma_{b}^{sam}$ and $\sigma_{b}^{gra}$ are introduced by $\rho_{GNDs}^{sam}$ and $\rho_{GNDs}^{gra}$, respectively.

**Figure 15:**
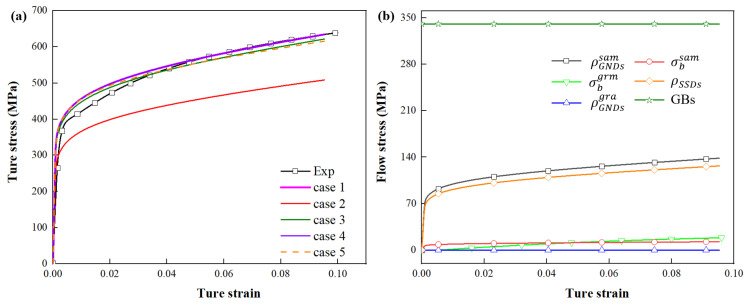
(**a**) True stress–strain versus predicted and (**b**) quantitative contributions of different factors to the flow stress of the DP steels.

Figure 15a shows the flow curves obtained from the simulations of the five conditions in Table 3. The flow stresses in case 1 and 4 are almost the same. The flow stresses in case 3 and 5 are slightly lower than that in case 1. Moreover, the flow stress in case 2 is much lower than that in case 1. It shows that $\rho_{GNDs}^{sam}$ contributes significantly to the work hardening of DP steel.

Figure 15b further quantifies the contribution of $\rho_{GNDs}^{sam}$, $\sigma_{b}^{sam}$, $\rho_{GNDs}^{gra}$, $\sigma_{b}^{gra}$ to the work hardening. It is found that the contributions of $\rho_{GNDs}^{gra}$ and $\sigma_{b}^{gra}$ to the work hardening are very low. The contribution of $\sigma_{b}^{sam}$ increases at first. At true strain equal to 0.5%, it reaches its maximum ~14 MPa. $\rho_{GNDs}^{sam}$ demonstrates a significant contribution to the work hardening. At true strain equal to 9.53%, it reaches its maximum ~140 MPa. With increasing strain, the strengthening effect of SSDs gradually increases to about 126 MPa. Since the grain size is assumed to be constant during the deformation process, the contribution of GBs is kept at about 340.5 MPa.

The quantitative estimation of the contribution of various strengthening mechanisms shows that YS is controlled by the Hall–Petch relationship, while the work hardening is controlled by $\rho_{SSDs}$, $\sigma_{b}^{sam}$ and $\rho_{GNDs}^{sam}$. During the entire process of tensile deformation of DP steel, the effect at the grain-level can be ignored.

## 5. Conclusions

Based on the Voronoi algorithm and interface layer control programming, the RVE of DP steel was established. Combined with the user-defined material model, a multi-strengthening mechanism was introduced to investigate the plastic formation of DP steel. The influence of the plastic strain gradient phenomenon on the mechanical response of the DP steel formation process was further determined. At the same time, the effects of DP distribution topology on the mechanical response of DP steel during plastic forming were obtained and discussed based on finite element simulation, the individual contributions of different strengthening mechanisms to the stress–strain response of DP steel were discussed in depth as well. The findings of this study are briefly summarized as follows:

1. The thickness of the interface layer inside ferrite has a great influence on the plastic flow of DP steel. Adding an interfacial layer will change the location of the maximum stress in the matrix but has little effect on the strain trend. The consistency is optimum when the interface layer thickness is 30%. With the addition of the interface layer, the peak value of GNDs appears at the boundary between the interface layer and ferrite and decreases gradually with the increase of layer thickness, but the value of GNDs near the boundary of martensite remains unchanged.
2. Increasing the number of layers makes the GNDs more widely distributed. However, the change in layer number hardly affects the macroscopic stress and strain of DP steel and the GND values at the interface layer–martensite boundary and the interface layer–ferrite boundary.
3. The increase in martensite volume fraction enhances the effective flow performance and strain localization of DP steel and gradually increases the value of GNDs at the boundary between martensite and interface layer.
4. At the sample level, there is non-uniform deformation and accumulation of GNDs, which plays a major role in the strain hardening of DP steel. The contribution of GNDs accumulated at the sample level to the strain hardening of DP steel is up to 47%. The low density of GNDs and the back stress caused by the strain gradient of the grain level has a small effect on the strain hardening and strengthening of DP steel.

## Figures and Tables

**Table 1:** Chemical composition and average segregation coefficients of DP600 steel.

Element	C	Si	Mn	P	S	Al
Composition, wt%	0.075	0.05	1.66	0.04	0.06	0.32
Segregation coefficient, (1/k)	-	0.18	1.32	-	-	0.67

**Table 2:** The mechanical parameters of the martensite and ferrite.

Parameter	Symbol	Martensite Value	Ferrite Value	Ref.
Modulus of elasticity (MPa)	*E*	387,400	203,300	
Poisson’ ratio	*ν*	0.3	0.3	[14]
Lattice friction stress (MPa)	$\sigma_{0}$	700	163	
Reference strain rate (S^−1^)	${\dot{\varepsilon }}_{ref}$	1	1	
Rate sensitively exponent	*m*	20	20	
Hall–Petch constant (MPa·μm^1/2^)	$k_{HP}$	90	90	[46]
Taylor factor	*M*	3.06	3.06	[47]
Magnitude of Burgers vector (nm)	*b*	0.248	0.248	
Taylor constant	*α*	0.3	0.3	
Nye-factor	$\bar{r}$	1.9	1.9	[48]
Geometric factor	$k_{mfp}^{g}$	0.063	0.063	
Proportionality factor	$k_{mfp}^{dis}$	0.0085	0.0085	
Dynamic recovery constant 1	$k_{ann}^{0}$	1.5	1.5	
Dynamic recovery constant 2	$n_{0}$	21.0	21.0	
Pileup dislocations constant 1 (μm^−1^)	$k_{N}$	46	46	
Pileup dislocations constant 2	$N_{A}$	300	300	
Cut-off radius of the GNDs domain (μm)	*R*	3	3	
Initial dislocation density (m^−2^)	$\rho_{0}$	2 × 10^11^	2 × 10^11^	
Pileup factor related to grain size(μm^−1^)	*λ*	3.78	3.78	
Correction parameter of pileup dislocations	$N_{extra}$	0.62	0.62	
Grain size (μm)	*d*	8.4 ± 6.1	2.7 ± 1.6	
Reference grain size (μm)	$d_{ref}$	8.4	2.7	

**Table 3:** Investigation of the individual contribution of different strengthening factors through modeling cases.

Case	$\rho_{GNDs}^{sam}$	$\sigma_{b}^{sam}$	$\rho_{GNDs}^{gra}$	$\sigma_{b}^{gra}$
Case 1	√	√	√	√
Case 2	×	√	√	√
Case 3	√	×	√	√
Case 4	√	√	×	√
Case 5	√	√	√	×
